# Development of a CRISPR/Cas9 System for Methylococcus capsulatus *In Vivo* Gene Editing

**DOI:** 10.1128/AEM.00340-19

**Published:** 2019-05-16

**Authors:** Timothy Tapscott, Michael T. Guarnieri, Calvin A. Henard

**Affiliations:** a National Bioenergy Center, National Renewable Energy Laboratory (NREL), Golden, Colorado, USA; North Carolina State University

**Keywords:** CRISPR/Cas9, Methylococcus capsulatus, gene editing, methane biocatalyst, methane monooxygenase, methanotroph

## Abstract

Methanotrophic bacteria play a crucial role in the Earth’s biogeochemical cycle and have the potential to be employed in industrial biomanufacturing processes due to their capacity to use natural gas- and biogas-derived methane as a sole carbon and energy source. Advanced gene-editing systems have the potential to enable rapid, high-throughput methanotrophic genetics and biocatalyst development. To this end, we employed a series of broad-host-range expression plasmids to construct a conjugatable clustered regularly interspaced short palindromic repeats (CRISPR)/Cas9 gene-editing system in Methylococcus capsulatus (Bath). Heterologous coexpression of the Streptococcus pyogenes Cas9 endonuclease and a synthetic single guide RNA (gRNA) showed efficient Cas9 DNA targeting and double-stranded DNA (dsDNA) cleavage that resulted in cell death. We demonstrated effective *in vivo* editing of plasmid DNA using both Cas9 and Cas9^D10A^ nickase to convert green fluorescent protein (GFP)- to blue fluorescent protein (BFP)-expressing cells with 71% efficiency. Further, we successfully introduced a premature stop codon into the soluble methane monooxygenase (sMMO) hydroxylase component-encoding *mmoX* gene with the Cas9^D10A^ nickase, disrupting sMMO function. These data provide proof of concept for CRISPR/Cas9-mediated gene editing in M. capsulatus. Given the broad-host-range replicons and conjugation capability of these CRISPR/Cas9 tools, they have potential utility in other methanotrophs and a wide array of Gram-negative microorganisms.

**IMPORTANCE** In this study, we targeted the development and evaluation of broad-host-range CRISPR/Cas9 gene-editing tools in order to enhance the genetic-engineering capabilities of an industrially relevant methanotrophic biocatalyst. The CRISPR/Cas9 system developed in this study expands the genetic tools available to define molecular mechanisms in methanotrophic bacteria and has the potential to foster advances in the generation of novel biocatalysts to produce biofuels, platform chemicals, and high-value products from natural gas- and biogas-derived methane. Further, due to the broad-host-range applicability, these genetic tools may also enable innovative approaches to overcome the barriers associated with genetically engineering diverse, industrially promising nonmodel microorganisms.

## Abstract (precis)

In this study, we targeted the development and evaluation of broad-host-range CRISPR/Cas9 gene-editing tools in order to enhance the genetic-engineering capabilities of an industrially relevant methanotrophic biocatalyst. The CRISPR/Cas9 system developed in this study expands the genetic tools available to define molecular mechanisms in methanotrophic bacteria and has the potential to foster advances in the generation of novel biocatalysts to produce biofuels, platform chemicals, and high-value products from natural gas- and biogas-derived methane. Further, due to the broad-host-range applicability, these genetic tools may also enable innovative approaches to overcome the barriers associated with genetically engineering diverse, industrially promising nonmodel microorganisms.

## INTRODUCTION

The clustered regularly interspaced short palindromic repeats (CRISPR)/CRISPR-associated protein 9 (Cas9) prokaryotic immune system from Streptococcus pyogenes has revolutionized genetic-engineering capabilities in a wide array of organisms. CRISPR/Cas9 gene editing is mediated by the programmable Cas9 endonuclease, which targets a genomic locus with high specificity and induces a double-stranded DNA (dsDNA) break (1–4). Target specificity is controlled by a coexpressed single guide RNA (gRNA) that contains a 20-base protospacer complementary to the target sequence, which is adjacent to a 5′-NGG-3′ protospacer-adjacent motif (PAM) site (2, 3).

Cas9-induced dsDNA breaks can result in repair via error-prone nonhomologous end joining (NHEJ) or high-fidelity homology-directed repair (HDR) (5). Although a subset of bacteria contain Ku- and DNA ligase-catalyzed NHEJ systems, most rely on RecA/RecBCD-dependent HDR to repair Cas9-induced dsDNA breaks (5, 6). In bacteria in which dsDNA breaks have been reported to be lethal, the Cas9^D10A^ nickase has been successfully employed for CRISPR-based gene editing (7–9). This variant contains a disabled RuvC1 nuclease domain that can induce single-stranded DNA (ssDNA) nicks in order to induce single-nick-assisted HDR (3, 7–9). In some microbes tested, Cas9^D10A^ mediated higher gene editing efficiencies than wild-type Cas9 (7–9). Combined, these CRISPR/Cas9 tools can enable robust multiplex and high-throughput gene-editing strategies (10–12) and may fast track the development of nonmodel microorganisms with limited genetic tractability for industrial applications.

Methanotrophic bacteria are key players in Earth’s biogeochemical carbon cycle and are of increasing industrial interest for their capacity to utilize methane as a sole carbon and energy source (13). The model gammaproteobacterial methanotroph Methylococcus capsulatus has been extensively studied for decades and is currently used for the industrial production of single-cell protein. Broad-host-range replicative plasmids containing RP4/RK2, RSF1010, and pBBR1 replicons are functional in M. capsulatus and have enabled the development of promoter-probe vectors and heterologous gene expression in the organism (14, 15). Further, chromosomal insertions and unmarked genetic mutations using allelic-exchange vectors and sucrose or p-chlorophenylalanine counterselection have been reported (15–19). These tools have served as a basis for the recent expansion of the methanotroph genetic toolbox that has enabled several proteobacterial methanotrophs to be engineered to convert methane-rich natural gas and aerobic-digestion-derived biogas into high-value products (20–25). Notably, these tools also lay the foundation for the development of advanced CRISPR genome-editing systems that facilitate multiplex or high-throughput gene-editing strategies. The development of advanced genome-editing tools offers a means to enable rapid evaluation of fundamental methanotrophic governing mechanisms while expanding metabolic engineering capabilities in these hosts for methane sequestration, bioremediation, and biomanufacturing.

In this study, we developed broad-host-range CRISPR/Cas9 gene-editing tools and evaluated their efficacy in the methanotroph M. capsulatus. Using the CRISPR/Cas9 system, we demonstrated editing of methanotroph-harbored plasmid DNA by introducing *in vivo* point mutations in a gene encoding green fluorescent protein (GFP) to generate a blue fluorescent protein (BFP) variant. Further, we successfully achieved chromosomal editing by generating a soluble methane monooxygenase (sMMO) mutant strain via the introduction of a premature stop codon in the *mmoX* open reading frame using the Cas9^D10A^ nickase. The CRISPR/Cas9 tools developed here will facilitate the development of advanced methanotrophic biocatalysts and have potential utility in an array of nonmodel, industrially promising bacteria.

## RESULTS AND DISCUSSION

### Development of a broad-host-range CRISPR/Cas9 gene-editing system.

**FIG 1:**
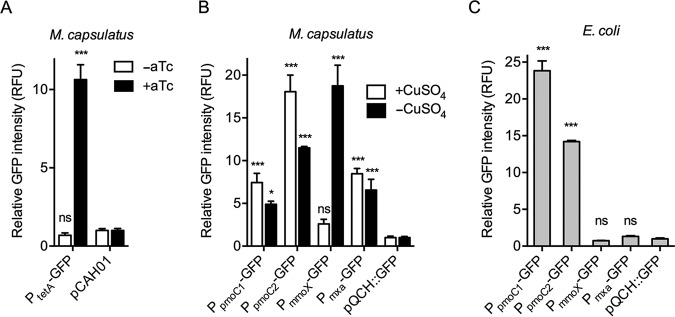
Promoter activity in M. capsulatus. (A) GFP fluorescence in M. capsulatus expressing GFP from the tetracycline promoter/operator (P*_tetA_*) in pCAH01::GFP. Where indicated, GFP was induced by plating on NMS agar supplemented with 500 ng/ml aTc for 72 h. The empty pCAH01 plasmid was used as a negative control. (B) GFP fluorescence in M. capsulatus with GFP expression controlled by the indicated gene promoters in pQCH::GFP. Fluorescence intensity was measured from cells grown on NMS agar with or without 5 µM CuSO_4_ for 72 h. (C) GFP fluorescence in E. coli expressing GFP from the indicated promoters in pQCH::GFP. Fluorescence intensity was measured from cells grown to an OD_600_ of 0.5 in LB liquid medium. In panels B and C, the promoterless pQCH::GFP plasmid was used as a negative control. The data represent the fluorescence intensity normalized to OD_600_ and are depicted as mean RFU and standard deviations (SD) from 3 independent replicates. ***, *P < *0.001; *, *P < *0.05; ns, not significant.

**FIG 2:**
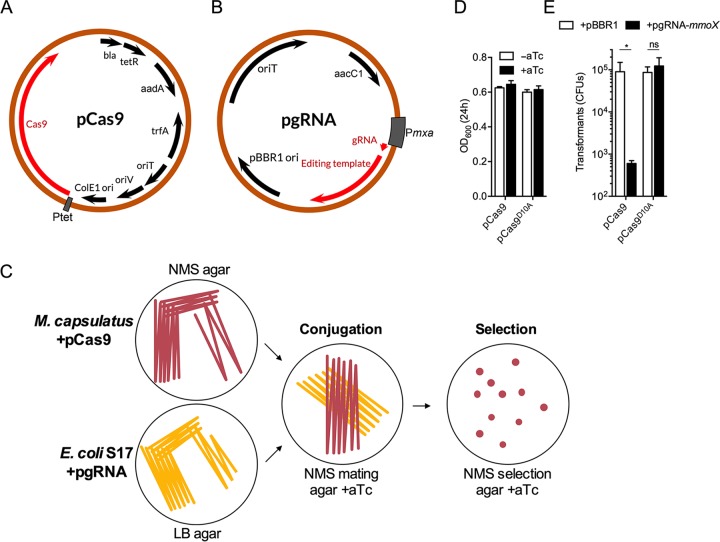
Broad-host-range CRISPR/Cas9 gene-editing system. (A) Plasmid map of pCAH01Sp^R^::Cas9 (pCas9). Inducible expression of Cas9 is driven from the tetracycline promoter/operator (P*_tetA_*). (B) Plasmid map of pBBR1-gRNA (pgRNA) containing a 1-kb DNA repair template. Expression of the gRNA is driven from the M. capsulatus *mxaF* promoter (P*_mxa_*). (C) Experimental design schematic of the CRISPR/Cas9 gene-editing system. M. capsulatus harboring pCas9 was conjugated with E. coli S17 harboring pgRNA on NMS mating agar supplemented with 500 ng/ml aTc. After 48 h of conjugation, the biomass was spread onto NMS selection agar containing 500 ng/ml aTc, gentamicin, and spectinomycin until colonies appeared. (D) OD_600_ of bacterial cultures 24 h postinoculation with (+aTc) or without (−aTc) Cas9 or Cas9^D10A^ induction. The cultures were inoculated at an OD_600_ of 0.1. (E) CFU of Cas9- or Cas9^D10A^-expressing M. capsulatus after conjugation with pgRNA-*mmoX*. Empty pBBR1 plasmid was used as a negative control. The data are depicted as mean CFU and SD from 3 independent replicates. *, *P < *0.05; ns, not significant.

We employed the superfolder GFP (26) reporter to evaluate the functionality and strength of heterologous and native M. capsulatus promoters to be used for expression of Cas9 nuclease and gRNA components. We tested whether pCAH01, an RK2-based broad-host-range expression plasmid that contains the inducible tetracycline promoter/operator (P*_tetA_*), previously demonstrated to function in the related gammaproteobacterial methanotroph Methylomicrobium buryatense 5GB1 (21, 23), was also functional in M. capsulatus. The P*_tetA_* promoter exhibited strong inducible activation in M. capsulatus, as indicated by an ∼10-fold increase of GFP fluorescence in pCAH01::GFP-harboring cells after exposure to the anhydrotetracycline (aTc) inducer (Fig. 1A). Based on the ability to temporally control gene expression, Cas9- and the Cas9^D10A^ nickase variant-encoding genes were cloned downstream of P*_tetA_* in pCAH01Sp^R^ to generate pCas9 (Fig. 2A) and pCas9^D10A^, respectively (see Fig. S1A in the supplemental material).

Next, we evaluated the activity of M. capsulatus promoters to select a suitable promoter to drive constitutive gRNA expression. Promoters from the gamma subunit of particulate methane monooxygenase 1 (P*_pmoC1_*), particulate methane monooxygenase 2 (P*_pmoC2_*), the sMMO hydroxylase component *mmoX* (P*_mmoX_*), and methanol dehydrogenase *mxaF* (P*_mxa_*) genes were cloned upstream of a GFP coding sequence in the RSF1010-derived vector pQCH (see Fig. S1B), and promoter activity was assessed via fluorescence. As previously demonstrated via transcriptomic studies (19, 27, 28), P*_pmoC1_*, P*_pmoC2_*, and P*_mxa_* were highly active in M. capsulatus, driving significant GFP transcription under both copper-replete and copper-depleted conditions (Fig. 1B). As expected, we detected limited P*_mmoX_* promoter activity under copper-replete growth conditions; however, an ∼7.5-fold increase in GFP expression was observed when cells were grown under copper-depleted conditions (Fig. 1B) (19, 27, 28). Notably, the copper switch-regulated P*_mmoX_* promoter may be employed for the inducible expression of heterologous genes in M. capsulatus. Both P*_pmoC1_* and P*_pmoC2_* were functional in Escherichia coli, but, interestingly, P*_mxa_* and P*_mmoX_* promoter activity was not detected in the organism (Fig. 1C), presumably due to the absence of required regulatory factors unique to M. capsulatus (15). Based on these results, we chose the P*_mxa_* promoter to express the gRNA due to the undetectable levels of GFP expression from this promoter in E. coli in order to avoid gRNA expression during cloning or conjugation procedures. A schematic of the broad-host-range plasmid pBBR1 containing a P*_mxa_*-expressing gRNA and a 1-kb DNA repair template (pgRNA) is depicted in Fig. 2B.

Initial experiments evaluating Cas9 or Cas9^D10A^ expression in M. capsulatus indicated that nuclease expression did not affect bacterial growth in the absence of gRNA expression (Fig. 2D). Next, Cas9 or Cas9^D10A^ was coexpressed with an *mmoX*-targeting gRNA to evaluate nuclease targeting and activity using cell viability as a phenotypic readout. Transformants coexpressing Cas9 and pgRNA-*mmoX* exhibited ∼99% cell death compared to cells expressing Cas9 without a gRNA (Fig. 2E). The surviving ∼1% represent the background of the system and may serve as a putative limiting factor for gene-editing efficiency (29). Conversely, DNA digestion by Cas9^D10A^ did not result in cell death (Fig. 2E) and, assuming Cas9^D10A^ is functional under these experimental conditions, indicates that dsDNA breaks have a high degree of lethality while ssDNA nicks are more efficiently repaired by native M. capsulatus systems.

### *In vivo* plasmid editing.

**FIG 3:**
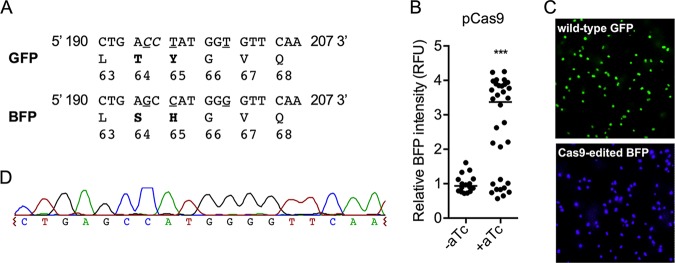
CRISPR/Cas9-targeted editing of plasmid DNA converting GFP to BFP. (A) Amino acid (boldface) and nucleotide (underlined) substitutions required to convert GFP to BFP with the Cas9 PAM site (italicized). (B) BFP intensity of Cas9-expressing M. capsulatus after conjugation with pgRNA-GFP^BFP^. Where indicated, Cas9 was induced by plating on NMS agar supplemented with 500 ng/ml aTc during mating and selection. Each data point represents the fluorescence intensity of a unique colony as RFU normalized to the OD_600_. The horizontal line represents the median fluorescence intensity. (C) Representative fluorescence micrograph of GFP-expressing and Cas9-edited BFP-expressing M. capsulatus. (D) Representative sequencing chromatogram of a Cas9-edited pQCH::P*_pmoC1_*-BFP locus. ***, *P < *0.001.

To evaluate CRISPR/Cas9 gene editing in M. capsulatus, we employed a screening assay with a direct fluorescent readout that shifts the emission and excitation of GFP to that of BFP following targeted gene editing (30). We constructed a vector (pgRNA-GFP^BFP^) with a *gfp*-targeting gRNA and a 1-kb DNA repair template containing the GFP point mutations 194C→G, 196T→C, and 201T→G, which introduces the missense codon substitutions T64S and Y65H to convert GFP to BFP while concurrently abolishing the Cas9 PAM site (Fig. 3A; see Fig. S2 in the supplemental material). No detectable BFP fluorescence, only GFP fluorescence, was observed in cells expressing pgRNA-GFP^BFP^ and GFP, demonstrating that the native homologous-recombination machinery did not integrate the DNA repair template into the *gfp* locus in the absence of Cas9 and that BFP is not expressed from the pgRNA-GFP^BFP^ repair template (Fig. 3B). Positive transformants coexpressing Cas9, pgRNA-GFP^BFP^, and GFP were analyzed for BFP fluorescence to determine editing efficiency. In the absence of Cas9 induction, we observed ∼5% BFP-positive transformants after selection (Fig. 3B), indicating that leaky Cas9 expression from P*_tetA_* is sufficient for gene editing. The induction of Cas9 during the conjugal transfer of pgRNA-GFP^BFP^ significantly increased plasmid DNA-editing efficiency, with 71% of the transformants originally encoding GFP now expressing BFP (Fig. 3B). Fluorescence microscopy of isolated BFP-expressing colonies showed that all the cells uniformly expressed BFP, with no GFP-expressing cells observed in the colony population (Fig. 3C). Sequence analysis of the fluorescent-protein-encoding loci from transformants identified as BFP positive confirmed the incorporation of the p.T64S and p.Y65H GFP-to-BFP mutations (Fig. 3D).

**FIG 4:**
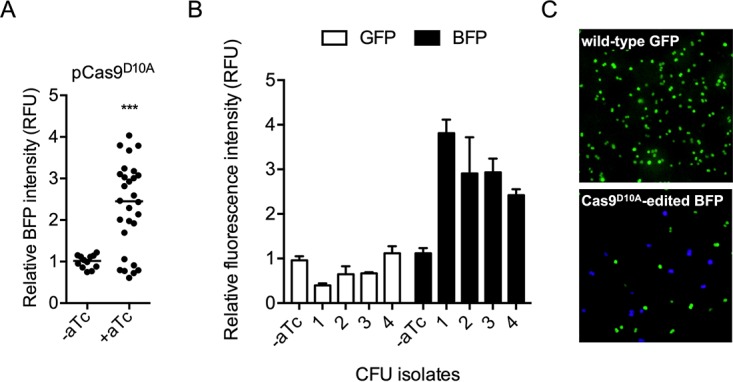
CRISPR/Cas9^D10A^ nickase-targeted editing of plasmid DNA converting GFP to BFP. (A) BFP fluorescence in Cas9^D10A^-expressing M. capsulatus after conjugation with pgRNA-GFP^BFP^. Where indicated, Cas9^D10A^ was induced by plating on NMS agar supplemented with 500 ng/ml aTc during mating and selection. Each data point represents the fluorescence intensity of a unique colony as RFU normalized to the OD_600_. The horizontal line represents the median fluorescence intensity. (B) BFP and GFP fluorescence in representative transformants coexpressing Cas9^D10A^ and pgRNA-GFP^BFP^. The data represent mean RFU and SD from 3 independent replicates. (C) Representative fluorescence micrograph of Cas9^D10A^-edited BFP-expressing M. capsulatus cells. ***, *P < *0.001.

Similar to the experimental design with Cas9 described above, we tested whether the Cas9^D10A^ nickase could also be utilized for targeted DNA editing. In the absence of Cas9^D10A^ induction, no BFP fluorescence was detected in pgRNA-GFP^BFP^-expressing transformants (Fig. 4A). However, when Cas9^D10A^ and pgRNA-GFP^BFP^ were coexpressed, we found that 71% of the transformants exhibited BFP fluorescence at varying intensities (Fig. 4A). Intriguingly, many BFP-expressing transformants were also positive for GFP fluorescence, with the degree of BFP fluorescence intensity positively correlated with a decrease in GFP fluorescence intensity (Fig. 4B). Fluorescence microscopy showed that transformant colonies consisted of both GFP- and BFP-expressing cells (Fig. 4C), presumably due to the expansion of plasmid copies that either were not cleaved by Cas9^D10A^ or were repaired after cleavage without incorporation of the homologous DNA repair template.

### *In vivo* genome editing.

**FIG 5:**
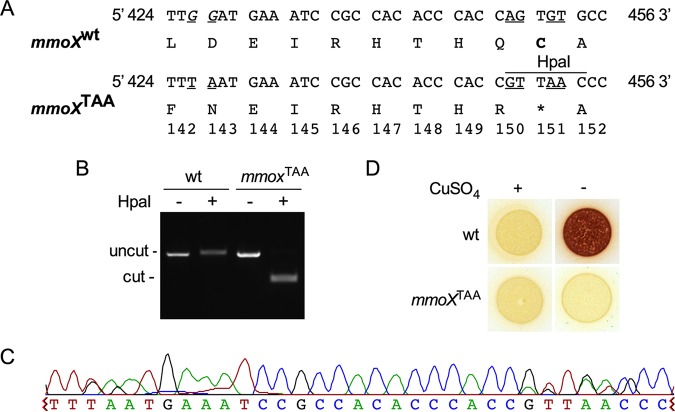
CRISPR/Cas9^D10A^-mediated editing of the M. capsulatus sMMO hydroxylase *mmoX* chromosomal locus. (A) Nonsense codon (boldface) and nucleotide (underlined) substitutions for generation of the *mmoX* p.C151X (*mmoX*^TAA^) mutation with the Cas9 PAM site shown (italicized). wt, wild type. (B) Representative agarose gel showing HpaI-digested *mmoX* PCR products from the wild type or a positive *mmoX*^TAA^ transconjugant. (C) Representative sequencing chromatogram of a Cas9^D10A^-edited *mmoX*^TAA^ locus. (D) Wild-type and *mmoX*^TAA^ biomass on NMS agar with or without 5 µM CuSO_4_. The activity of sMMO was assessed by naphthalene and *o*-dianisidine colorimetric assay. Positive sMMO activity is indicated by development of red coloration.

We next evaluated Cas9- and Cas9^D10A^-mediated chromosomal editing by targeting the *mmoX* gene encoding the sMMO hydroxylase component. We constructed a vector that harbored an *mmoX*-targeting gRNA and a DNA repair template containing an HpaI endonuclease restriction site, GTTAAC, which concurrently introduces a nonsense *mmoX* C151X (*mmoX*^TAA^) mutation (pgRNA-*mmoX*^TAA^) (Fig. 5A; see Fig. S3 in the supplemental material). Transformants coexpressing pgRNA-*mmoX*^TAA^ and Cas9 or Cas9^D10A^ were screened by colony PCR and subsequent digestion with HpaI endonuclease. We were unable to isolate an *mmoX*^TAA^ transformant in Cas9- and pgRNA-*mmoX*^TAA^-expressing tranformants. In contrast, we identified the targeted *mmoX*^TAA^ edit in 2% (5/250) of the Cas9^D10A^ nickase- and pgRNA-*mmoX*^TAA^-expressing transformants, as verified by HpaI digestion (Fig. 5B). Incorporation of the *mmoX*^TAA^ nonsense mutation into the chromosome was also verified by sequence analysis (Fig. 5C). Further, the colorimetric sMMO activity assay demonstrated that the *mmoX*^TAA^ strain was unable to convert naphthalene to naphthol after copper-depleted growth, confirming disruption of sMMO functionality (Fig. 5D).

Inducing single-nick-assisted HDR by the Cas9^D10A^ nickase has been employed in other microbes when wild-type Cas9-based editing has been unsuccessful (7–9). Previous studies have demonstrated that single-nick-assisted HDR undergoes repair via an independent mechanism with higher fidelity than dsDNA break-induced repair (31). Our data indicate that the Cas9^D10A^-mediated nick induces higher chromosome recombination efficiency than a wild-type Cas9 dsDNA break, leading to increased numbers of transformants and enhanced *mmoX* locus-editing efficiency (Fig. 2D and 5). Intriguingly, contrary to the differential observed for chromosomal editing, plasmid-editing efficiencies were identical whether Cas9 or Cas9^D10A^ was utilized. Future studies are thus needed to understand the differences in plasmid- and chromosome-editing efficiencies observed during the course of these investigations. Notably, successful incorporation of exogenous DNA into plasmid or genomic DNA by M. capsulatus was achieved via native recombination machinery. Evaluating the efficacy of heterologous recombinases, such as those employed in lambda red recombineering, may offer a potential means to enhance Cas9-mediated editing while also decreasing repair template size requirements (32, 33).

### Conclusions.

Rational metabolic-engineering pursuits in methanotrophic bacteria require several metabolic mutations in order to enhance metabolic flux, end products, stress tolerances, and substrate utilization for the improved production of bio-based products (20). The tools developed here may enable CRISPR-based multiplex gene knockout strategies that can accelerate this time- and labor-intensive process. Further, CRISPR-based gene editing, together with high-throughput oligonucleotide synthesis, presents a path to genetic-library construction targeting rate-limiting metabolic enzymes to isolate strains with enhanced or altered characteristics (10, 12). The ability to utilize Cas9 for DNA targeting makes it possible to also leverage the suite of available Cas9 variants (e.g., dCas9) (34–41) for transcriptional control, development of novel regulatory circuits, or optimization of CRISPR/Cas editing efficiency to enable advanced synthetic biology applications in methanotrophic bacteria.

In this study, we developed dual-plasmid, broad-host-range CRISPR/Cas9 tools and demonstrated targeted plasmid and chromosomal DNA editing with Cas9 and Cas9^D10A^ in the methanotroph M. capsulatus. This genetic system represents an advance in methanotroph molecular microbiology via expansion of the genetic toolbox. These advanced genetic tools may facilitate innovative strain engineering strategies that enable the development of methanotrophic biocatalysts for the production of biofuels, platform chemicals, and high-value products from methane. Further, novel molecular mechanisms underlying methanotroph biology can be probed with the addition of CRISPR/Cas9 to the methanotroph genetic toolbox. The replicons utilized in the CRISPR/Cas9 system developed here are recognized by phylogenetically diverse bacteria; thus, they have the potential to facilitate facile genetic querying and innovative strain-engineering strategies for the development of industrial biocatalysts in an array of nonmodel microbes.

## MATERIALS AND METHODS

### Bacterial strains and cultivation conditions.

The strains used in this study are described in Table 1. Methylococcus capsulatus (Bath) was cultured on modified nitrate mineral salts (NMS) agar supplemented with 5 µM CuSO_4_ (the formulation is shown in Table S1 in the supplemental material), unless otherwise indicated, at 37°C inside stainless-steel gas chambers (Schuett-biotec GmbH) containing 20% (vol/vol) methane in air (42). The NMS agar was supplemented with 100 µg/ml kanamycin, 100 µg/ml spectinomycin, and/or 30 µg/ml gentamicin for selection and cultivation of the respective M. capsulatus strains. E. coli strains were cultured on lysogeny broth (LB) agar or in LB liquid medium at 37°C at 200 rpm. LB liquid medium was supplemented with 50 µg/ml kanamycin, 50 µg/ml spectinomycin, 10 µg/ml gentamicin, and/or 100 µg/ml ampicillin for selection and cultivation of the respective E. coli strains. Broad-host-range plasmids were transferred to M. capsulatus via biparental mating using E. coli S17-1 cells on NMS mating agar (the formulation is shown in Table S1), as described previously (25). Prior to conjugation, M. capsulatus biomass harboring pCas9 or pCas9^D10A^ was spread on NMS mating medium supplemented with 500 ng/ml aTc and incubated at 37°C inside stainless-steel gas chambers (Schuett-biotec GmbH) containing 20% (vol/vol) methane (99.97% purity) in air for 24 h. A schematic of the experimental design for using the CRISPR/Cas9 system in M. capsulatus is shown in Fig. 2C.

**TABLE 1:** Strains and plasmids

Name	Genotype or description	Source
Strains		
Methylococcus capsulatus Bath	Wild type	ATCC 33009
E. coli Zymo 10B	F^−^ *mcrA* Δ(*mrr-hsdRMS-mcrBC*) Φ80*lacZ*ΔM15 Δ*lacX74 recA1 endA1 araD139* Δ(*ara leu*) 7697 *galU galK rpsL nupG* λ^−^	Zymo Research
E. coli S17-1	Tp^r^ Sm^r^ *recA thi pro hsd*(r^−^ m^+^)RP4-2-Tc::Mu::Km Tn*7*	ATCC 47055
Plasmids		
pCAH01	P*_tetA_ bla tetR* CoE1 *ori* F1 *oriV oriT trfA ahp*	21
pCAH01Sp^R^	*ahp CDS* exchanged with *aadA CDS*	This study
pCAH01::GFP	GFP cloned downstream of P*_tetA_*	21
pCAH01Sp^R^::Cas9	Cas9 cloned downstream of P*_tetA_*	This study
pAWP78	Source of *ahp* locus used to generate pQCH	25
RSF1010	*oriV oriT mobABC repABC sul2 strAB*	44
pQCH	RSF1010Δ[7.626-2.200 kb] *ahp*	This study
pQCH::GFP	pQCH with promoterless superfolder GFP	This study
pQCH::P*_mmoX_*-GFP	P*_mmoX_* cloned upstream of GFP in pQCH::GFP	This study
pQCH::P*_mxa_*-GFP	P*_mxa_* cloned upstream of GFP in pQCH::GFP	This study
pQCH::P*_pmoC1_*-GFP	P*_pmoC1_* cloned upstream of GFP in pQCH::GFP	This study
pQCH::P*_pmoC2_*-GFP	P*_pmoC2_* cloned upstream of GFP in pQCH::GFP	This study
pQCH::P*_pmoC1_*-BFP	P*_pmoC1_*-BFP cloned from P*_pmoC1_*-GFP	This study
pBBR1MCS-5	pBBR *oriT aacC1*	45
pBBR1-GFP	P*_mxa_*-gRNA-GFP cloned into pBBR1	This study
pBBR1-GFP^BFP^	P*_mxa_*-gRNA-GFP^BFP^ cloned into pBBR1	This study
pBBR1-*mmoX*	P*_mxa_*-gRNA-*mmoX* cloned into pBBR1	This study
pBBR1-*mmoX*^TAA^	P*_mxa_*-gRNA-*mmoX*^TAA^ cloned into pBBR1	This study

To evaluate promoter activity, M. capsulatus harboring GFP reporter plasmids was spread onto NMS agar supplemented with 0 µM or 5 µM CuSO_4_. GFP expression from the tetracycline promoter/operator (P*_tetA_*) in pCAH01 was induced by plating on NMS agar supplemented with 500 ng/ml aTc. Strains were incubated at 37°C inside stainless-steel gas chambers (Schuett-biotec GmbH) in 20% (vol/vol) methane in air for 72 h, and the GFP fluorescence intensity was quantified as described below. Promoter activity was determined in E. coli subcultured 1/100 in LB liquid medium and incubated for ∼3 h to an optical density at 600 nm (OD_600_) of 0.5 at 37°C at 200 rpm. A 200-µl volume of E. coli cell suspension was transferred to a 96-well plate for quantification of the GFP fluorescence intensity as described below.

### Cloning and genetic manipulation.

The plasmids used in this study are described in Table 1. The primers and synthetic DNA fragments used in this study were synthesized by Integrated DNA Technologies, Inc. (IDT), and are described in Table 2 and Table S2 in the supplemental material, respectively. Plasmids and DNA inserts were amplified using Q5 High-Fidelity 2× Master Mix (NEB), assembled using Gibson NEBuilder HiFi DNA assembly (New England Biolabs), and transformed into Mix and Go competent E. coli strain Zymo 10B (Zymo Research), according to the manufacturers’ instructions. Genetic constructs were verified by Sanger sequencing (Genewiz). The Cas9 open reading frame was amplified, using primers TT16 and CAH537 (Table 2), from Addgene plasmid no. 42876 (29) and cloned into pCAH01Sp^R^ via Gibson assembly. The Cas9^D10A^ nickase variant was generated by site-directed mutagenesis with primers TT143 and TT144 (Table 2) using the QuikChange primer design program and protocol (Agilent). Single gRNAs containing a 20-mer adjacent to the PAM site on the target DNA and editing cassettes were synthesized by IDT (see Table S2) and cloned into the pBBR1MCS-5 vector via Gibson assembly. pQCH was constructed by replacing a 3,312-bp region of RSF1010 containing the *sulfR*, *smrA*, and *smrB* genes with the *kan2* locus from pAWP78 (25). To evaluate promoter activity, native M. capsulatus promoters were cloned upstream of the superfolder green fluorescent protein-encoding gene (26) into pQCH.

**TABLE 2:** Primers

Purpose	Primer name^*a*^	Sequence^*b*^
Insert *aadA* into pCAH01	CAH520 *aadA* F	cagtgttacaaccaattaaccaattctgatTTATTTGCCGACTACCTTG
	CAH521 *aadA* R	cttacataaacagtaatacaaggggtgttaATGGCTTGTTATGACTGTTTTTTTG
	CAH522 pCAH01 F	TAACACCCCTTGTATTACTG
	CAH519 pCAH01 R	ATCAGAATTGGTTAATTGGTTG
Clone Cas9 into pCAH01	TT16 Cas9 F	cactccctatcagtgatagagaaaagtgaaATGGATAAGAAATACTCAATAGGC
	CAH537 Cas9 R	cttcacaggtcaagcttTTTTAGGAGGCAAAAATGGATAAG
	CAH152 pCAH01 F	AAGCTTGACCTGTGAAGTG
	CAH149 pCAH01 R	TTCACTTTTCTCTATCACTGATAG
Construct Cas9^D10A^	TT143 Cas9^D10A^ F	GAAATACTCAATAGGCTTAGCCATCGGCACAAATAGCGTCG
	TT144 Cas9^D10A^ R	CGACGCTATTTGTGCCGATGGCTAAGCCTATTGAGTATTTC
Construct pQCH	CAH1032 *ahp* F	ttatattcaatggcttatttGCTCGGGACGCACGGCGC
	CAH1033 *ahp* R	cggaacatgcctcatgtggcGCGTGATCTGATCCTTCAACTCAGCAAAAGTTCGATTTAT
	CAH1034 RSF1010 F	GCCACATGAGGCATGTTCCG
	CAH1031 RSF1010 R	AAATAAGCCATTGAATATAAAAGATAAAAATGTC
Construct pQCH::GFP	CAH1044 pQCH F	CATACAGTCTATCGCTTAGCG
	CAH1037 pQCH R	TATTGCAAGGACGCGGAAC
	CAH1045 GFP F	ATGAGCAAAGGAGAAGAAC
Amplify pQCH::GFP parts	CAH1040 GFP F	gaggaaacaagtaATGAGCAAAGGAGAAGAAC
	CAH1041 GFP R	ttatttgatgcctTTATTTGTAGAGCTCATCC
	CAH1042 rrnBT1T2 F	gctctacaaataaAGGCATCAAATAAAACGAAAGGC
	CAH1043 rrnBT1T2 R	tttccgctaagcgatagactgtatgCATCCGTCAGGATGGCCTTC
Clone promoters into pQCH::GFP	CAH1046 P*_mxa_* F	aggcatgttccgcgtccttgcaataGAGGTTCAGGCGAAACCG
	CAH1047 P*_mxa_* R	ctcctttgctcatGTGTCTCCTCCAAGAATGATTG
	CAH1048 P*_pmoC1_* F	aggcatgttccgcgtccttgcaataAACGTCACGATGGGTGTTC
	CAH1049 P*_pmoC1_* R	ctcctttgctcatTGTTTGTTCCTCCTAAAGTGATG
	CAH1050 P*_pmoC2_* F	aggcatgttccgcgtccttgcaataCCCTCGTGTCCGGCGTAC
	CAH1051 P*_pmoC2_* R	ctcctttgctcatTTTTACCTCCAACTGTTATATCGATGTGAACAC
	CAH1038 P*_mmoX_* F	aggcatgttccgcgtccttgcaataTCCGCAGTGGTCGGATCG
	CAH1039 P*_mmoX_* R	ctcctttgctcatTACTTGTTTCCTCCGTAACACATTCTATG
Construct pgRNA-GFP and pgRNA-*mmoX*	TT254 P*_mxa_* gRNA F	tccaattcgccctatagtgaGAGGTTCAGGCGAAACCG
	TT272 gRNA only R	gcaatagacataagcggctaGGATCAGATCACGCATCTTC
	TT256 pBBR1 F	TAGCCGCTTATGTCTATTGCTG
	TT253 pBBR1 R	TCACTATAGGGCGAATTGGAG
Construct GFP^BFP^ editing template	TT207 GFP F	atctgatccttcggaccgacggattGGACCGACGGATTTTATG
	TT208 BFP R	cattgaaccccatggcTCAGAGTAGTGACAAGTGTTG
	TT209 BFP F	ctactctgagccatgggGTTCAATGCTTTTCCCGTT
	TT255 GFP R	gcaatagacataagcggctaTGCCATGTGTAATCCCAG
Check GFP/BFP editing locus	TT288 GFP check F	TCCGCGTCCTTGCAATAAAC
	TT289 GFP check R	CCGCTAAGCGATAGACTGTATG
	TT271 GFP seq R	GTACATAACCTTCGGGCATG
Check *mmoX* editing locus	TT290 *mmoX* check F	CCAGTACGTCACCGTTATG
	TT291 *mmoX* check R	AGATCTTGCCGTAGTGGTC
	TT292 *mmox* seq F	CTGGAAGTGGGCGAATAC

a F, forward; R, reverse.

b Lowercase indicates homologous sequence for Gibson assembly.

### GFP and BFP expression quantification.

To evaluate Cas9- and Cas9^D10A^-mediated plasmid editing, fluorescence intensity was measured in a Fluostar Omega microplate reader (BMG Labtech) at an excitation wavelength (λ_ex_) of 485 nm and an emission wavelength (λ_em_) of 520 nm (GFP) or a λ_ex_ of 355 nm and a λ_em_ of 460 nm (BFP). For GFP-to-BFP gene-editing experiments, the data represent relative fluorescence units (RFU) of the measured BFP intensity relative to pQCH::P*_pmoC1_*-GFP control intensity normalized to the cell density.

### Verification of mutations by colony PCR and HpaI digestion.

To evaluate Cas9- and Cas9^D10A^-mediated genomic editing of the *mmoX* locus, colony PCR was performed with primers TT290 and TT291 (Table 2) using *Taq* 2× Master Mix (NEB) according to the manufacturer’s instructions. The PCR mixture was used directly as the template for HpaI endonuclease (NEB) digestion, and the edited strains were identified by positive DNA digestion visualized by DNA electrophoresis. Targeted editing of the *mmoX* locus in positive transformants was verified by sequence analysis.

### Colorimetric sMMO assay.

M. capsulatus sMMO activity was tested with a colorimetric assay as previously described (43). Briefly, ∼1e6 cells were spotted onto NMS agar with or without 5 µM CuSO_4_ and cultured at 37°C. After 96 h of growth, ∼300 to 400 mg naphthalene (Sigma-Aldrich) crystals was placed into the petri dish lid and incubated with the bacteria for 1 h at 37°C to allow conversion of naphthalene to naphthol. After incubation, 20 µl of freshly prepared 5-mg/ml *o*-dianisidine (Sigma-Aldrich), which turns purple in the presence of naphthol, was added directly to the M. capsulatus biomass. Color development was allowed to occur for 15 min at 37°C.

### Statistical analysis.

Statistical analysis of data was performed and graphical representations were created using GraphPad Prism 6.0 software. Determination of statistical significance between two comparisons was achieved using an unpaired *t* test. Determination of statistical significance between multiple comparisons was achieved using a one-way analysis of variance (ANOVA) followed by Dunnett’s test with the appropriate controls. Normal distribution and equal variance between test groups were assumed prior to performing statistical tests using Prism software.

## Supplementary Material

Supplemental file 1
