# Supplementary material for: Development of a CRISPR/Cas9 System for Methylococcus capsulatus In Vivo Gene Editing
Source: Appl Environ Microbiol. 2019 May 16;85(11):e00340-19. doi: 10.1128/AEM.00340-19 (PMC6532038; doi:10.1128/AEM.00340-19)
Supplement: Supplemental file 1 [file AEM.00340-19-s0001.pdf]

Supplemental Materials: Tapscott, *et al*.

Supplemental Figures

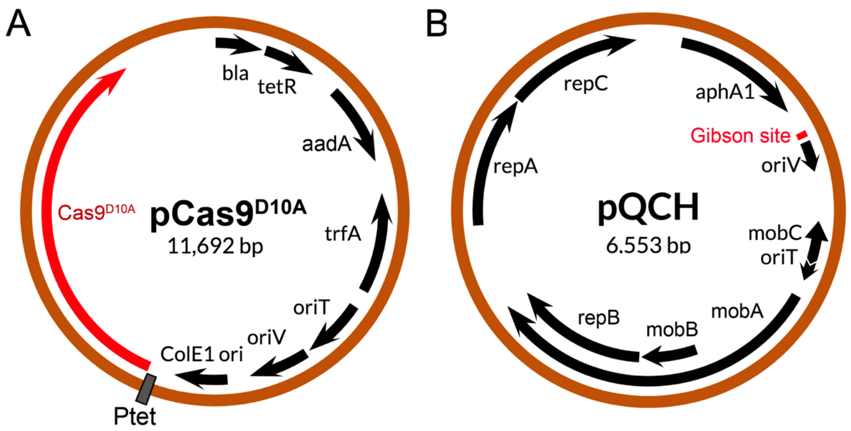

Figure S1. Development of CRISPR/Cas9 genetic tools
(A) Plasmid map of pCAH01::Cas9^{D10A} (pCas9^{D10A}). (B) Plasmid map of pQCH.

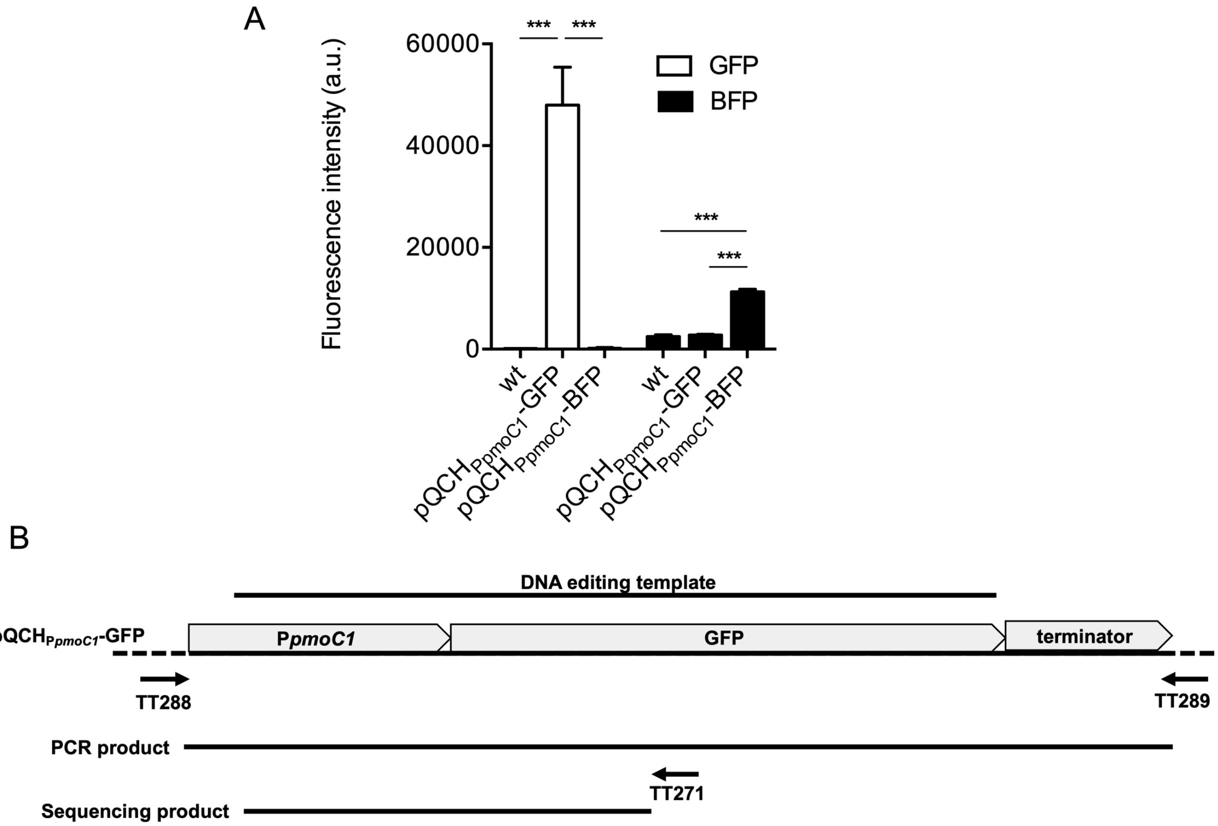

Figure S2. GFP to BFP editing

(A) Fluorescence intensity of *M. capsulatus* cells expressing pQCH::P$_{pmoC1}$-GFP and pQCH::P$_{pmoC1}$-BFP. Data depict mean arbitrary units (a.u.) ± S.D. from at least 3 unique CFUs (B) Schematic of pQCH::P$_{pmoC1}$-GFP locus that was targeted for gene editing. DNA editing template spanned 1 kb. For verification of genomic edits, primers TT288 and TT289 were used for genomic cPCR amplification and primer TT271 was used for Sanger sequencing. *** $p < 0.001$, ** $p < 0.01$, * $p < 0.05$, n.s. not significant.

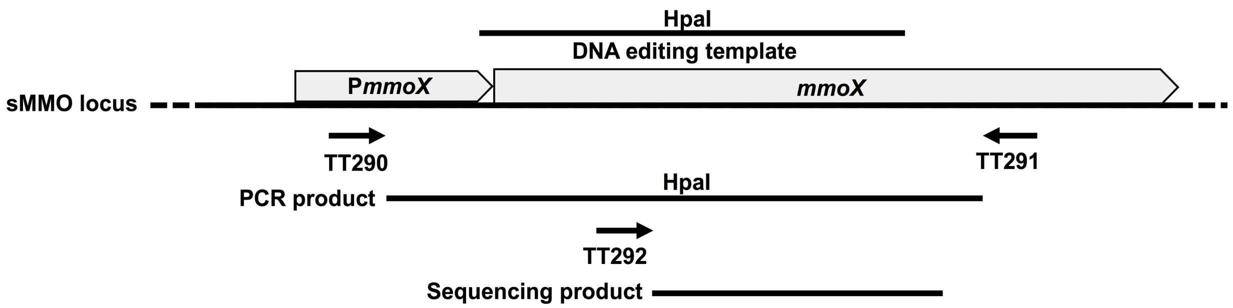

Figure S3. sMMO *mmoX* editing
Schematic of soluble methane monooxygenase (sMMO) hydroxylase component *mmoX* that was targeted for gene editing. DNA editing template spanned 1 kb of *mmoX* containing HpaI endonuclease recognition site and nonsense codon substitution p.C151X. For verification of genomic edits, primers TT290 and TT291 were used for genomic PCR amplification and primer TT292 was used for Sanger sequencing.

Supplemental Tables

Table S1. Media composition

Component	NMS	Mating
KNO_3	1 g/L	1 g/L
$MgSO_4$ x $7H_2O$	0.2 g/L	0.2 g/L
$CaCl_2$ x $2H_2O$	0.02 g/L	0.02 g/L
NaCl		2 g/L
Trace solution (1000x)	1 ml/L	1 ml/L
Nutrient Broth		1.2 g/L
Agar	15 g/L	15 g/L
Phosphate solution (pH 6.8)	50 m/L	50 m/L
**Phosphate solution (pH 6.8)**	g/L	
KH_2PO_4	5.44	
Na_2HPO_4	5.68	
**Trace mineral solution (1000x)**	g/L	
Na_2EDTA	5	
$FeSO_4$ x 7 H_2O	2	
$ZnSO_4$ x7 H_2O	0.3	
$MnCl_2$ x 4 H_2O	0.03	
$CoCl_2$ x 6 H_2O	0.2	
$CuSO_4$ x 5 H_2O	1.2	
Na_2O_4W x $2H_2O$	0.3	
$NiCl_2$ x 6 H_2O	0.05	
Na_2MoO_4 x 2 H_2O	0.05	
H_3BO_3	0.03	

Table S2. Gene fragments

Name / Sequence	Source
gRNA-GFP	IDT gBlock

GAGGTTCAGGCGAAACCGCAGACTCAAGGGCGCTTGCTCCCGGGAAAGATCGTATTAGTTTGCCTCGATCGGCGGTCCTT
GTGACAGGGAGATATTCCCGACGGATCCGGGGCATTCGAGCGGAACCGCCCGCCGTGGGAGTTTTTCCAGCGAGCATTCG
AGAGTTTTTCAAGGCGGCTTCGAGGGGTTATTCCGTAACGCCGCCGACATGATCTGTCCCAGAATCTCCGCCGCTGTTCG
TAGAGCGCCGATGCAGGGTCGGCATCAATCATTCTTGGAGGAGACACGGAAAAGCATTGAACACCATGTTTTAGAGCTAG
AAATAGCAAGTTAAAATAAGGCTAGTCCGTTATCAACTTGAAAAAGTGGCACCGAGTCGGTGCTTTTTTTTGTCGGGAAG
ATGCGTGATCTGATCC

gRNA-GFP & GFP^{BFP} editing template	PCR/Gibson

GAGGTTCAGGCGAAACCGCAGACTCAAGGGCGCTTGCTCCCGGGAAAGATCGTATTAGTTTGCCTCGATCGGCGGTCCTT
GTGACAGGGAGATATTCCCGACGGATCCGGGGCATTCGAGCGGAACCGCCCGCCGTGGGAGTTTTTCCAGCGAGCATTCG
AGAGTTTTTCAAGGCGGCTTCGAGGGGTTATTCCGTAACGCCGCCGACATGATCTGTCCCAGAATCTCCGCCGCTGTTCG
TAGAGCGCCGATGCAGGGTCGGCATCAATCATTCTTGGAGGAGACACGGAAAAGCATTGAACACCATGTTTTAGAGCTAG
AAATAGCAAGTTAAAATAAGGCTAGTCCGTTATCAACTTGAAAAAGTGGCACCGAGTCGGTGCTTTTTTTTGTCGGGAAG
ATGCGTGATCTGATCCTTCGGACCGACGGATTGGACCGACGGATTTTATGCAAGCATGTCGCTTTGACCAAGCCGGGATT
CCATGGAAGGGATGTCATCGGGAGAGTTATTTATGTCGTTGATTTATAAGAAACTACCCCTGCGTCAAAATGTCGCAGAT
TTTTCTTGACAGTTTGGGGGAGGGTGATAGACTCCCTCCACCGATGGACCGGTACCGCCTCTGTTGCGGGGTCCATGAAA
TGCCCGTTAGAGGCAGAACCGATAGGGAATTAGAGAAGCGGGCGTCGGCGCCGAATGCCGGCCCCTGTCAACCATCACTT
TAGGAGGAACAAACAATGAGCAAAGGAGAAGAACTTTTCACTGGAGTTGTCCCAATTCTTGTTGAATTAGATGGTGATGT
TAATGGGCACAAATTTTCTGTCCGTGGAGAGGGTGAAGGTGATGCTACAAACGGAAAACTCACCCTTAAATTTATTTGCA
CTACTGGAAAACTACCTGTTCCATGGCCAACACTTGTCACTACTCTGAGCCATGGGGTTCAATGCTTTTCCCGTTATCCG
GATCACATGAAACGGCATGACTTTTTCAAGAGTGCCATGCCCGAAGGTTATGTACAGGAACGCACTATATCTTTCAAAGA
TGACGGGACCTACAAGACGCGTGCTGAAGTCAAGTTTGAAGGTGATACCCTTGTTAATCGTATCGAGTTAAAAGGTATTG
ATTTTAAAGAAGATGGAAACATTCTCGGACACAAACTCGAGTACAACTTTAACTCACACAATGTATACATCACGGCAGAC
AAACAAAAGAATGGAATCAAAGCTAACTTCAAAATTCGCCACAACGTTGAAGATGGTTCCGTTCAACTAGCAGACCATTA
TCAACAAAATACTCCAATTGGCGATGGCCCTGTCCTTTTACCAGACAACCATTACCTGTCGACACAATCTGTCCTTTCGA
AAGATCCCAACGAAAAGCGTGACCACATGGTCCTTCTTGAGTTTGTAACTGCTGCTGGGATTACACATGGCA

gRNA-*mmoX*	IDT gBlock

GAGGTTCAGGCGAAACCGCAGACTCAAGGGCGCTTGCTCCCGGGAAAGATCGTATTAGTTTGCCTCGATCGGCGGTCCTT
GTGACAGGGAGATATTCCCGACGGATCCGGGGCATTCGAGCGGAACCGCCCGCCGTGGGAGTTTTTCCAGCGAGCATTCG
AGAGTTTTTCAAGGCGGCTTCGAGGGGTTATTCCGTAACGCCGCCGACATGATCTGTCCCAGAATCTCCGCCGCTGTTCG
TAGAGCGCCGATGCAGGGTCGGCATCAATCATTCTTGGAGGAGACACGATACAGCCATCGGACATAAGTTTTAGAGCTAG
AAATAGCAAGTTAAAATAAGGCTAGTCCGTTATCAACTTGAAAAAGTGGCACCGAGTCGGTGCTTTTTTTTGTCGGGAAG
ATGCGTGATCTGATCC

gRNA-*mmoX* & *mmoX*^{TAA} editing template	IDT gBlock

TCCAATTCGCCCTATAGTGAGAGGTTCAGGCGAAACCGCAGACTCAAGGGCGCTTGCTCCCGGGAAAGATCGTATTAGTT
TGCCTCGATCGGCGGTCCTTGTGACAGGGAGATATTCCCGACGGATCCGGGGCATTCGAGCGGAACCGCCCGCCGTGGGA
GTTTTTCCAGCGAGCATTCGAGAGTTTTTCAAGGCGGCTTCGAGGGGTTATTCCGTAACGCCGCCGACATGATCTGTCCC
AGAATCTCCGCCGCTGTTCGTAGAGCGCCGATGCAGGGTCGGCATCAATCATTCTTGGAGGAGACACCGGCTATCTGGCC
CAGGTGTGTTTTAGAGCTAGAAATAGCAAGTTAAAATAAGGCTAGTCCGTTATCAACTTGAAAAAGTGGCACCGAGTCGG
TGCTTTTTTTTGTCGGGAAGATGCGTGATCTGATCCGTCATCGGGCATTCATCATTCATAGAATGTGTTACGGAGGAAAC
AAGTAATGGCACTTAGCACCGCAACCAAGGCCGCGACGGACGCGCTGGCTGCCAATCGGGCACCCACCAGCGTGAATGCA
CAGGAAGTGCACCGTTGGCTCCAGAGCTTCAACTGGGATTTCAAGAACAACCGGACCAAGTACGCCACCAAGTACAAGAT
GGCGAACGAGACCAAGGAACAGTTCAAGCTGATCGCCAAGGAATATGCGCGCATGGAGGCAGTCAAGGACGAAAGGCAGT
TCGGTAGCCTGCAGGATGCGCTGACCCGCCTCAACGCCGGTGTTCGCGTTCATCCGAAGTGGAACGAGACCATGAAAGTG
GTTTCGAACTTCCTGGAAGTGGGCGAATACAACGCCATCGCCGCTACCGGGATGCTGTGGGATTCCGCCCAGGCGGCGGA
ACAGAAGAACGGCTATCTGGCCCAGGTGTTTAATGAAATCCGCCACACCCACCGTTAACCCTACGTCAACTACTACTTCG
CGAAGAACGGCCAGGACCCGGCCGGTCACAACGATGCTCGCCGCACCCGTACCATCGGTCCGCTGTGGAAGGGCATGAAG
CGCGTGTTTTCCGACGGCTTCATTTCCGGCGACGCCGTGGAATGCTCCCTCAACCTGCAGCTGGTGGGTGAGGCCTGCTT
CACCAATCCGCTGATCGTCGCAGTGACCGAATGGGCTGCCGCCAACGGCGATGAAATCACCCCGACGGTGTTCCTGTCGA
TCGAGACCGACGAACTGCGCCACATGGCCAACGGTTACCAGACCGTCGTTTCCATCGCCAACGATCCGGCTTCCGCCAAG
TATCTCAACACGGACCTGAACAACGCCTTCTGGACCCAGCAGAAGTACTTCACGCCGGTGTTGGGCATGCTGTTCGAGTA
TGGCTCCAAGTTCAAGGTCGAGCCGTGGGTCAAGACGTGGAACCGCTGGGTGTACGAGGACTGGGGCGGCATCTGGTAGC
CGCTTATGTCTATTGC
